# What is the impact of aerobic fitness and movement interventions on low-flow-mediated vasoconstriction? A systematic review of observational and intervention studies

**DOI:** 10.1177/1358863X211073480

**Published:** 2022-02-24

**Authors:** Myles W O’Brien, Jennifer L Petterson, Yanlin Wu, Nick W Bray, Derek S Kimmerly

**Affiliations:** 1 Division of Kinesiology, Dalhousie University, Halifax, NS, Canada; 2 Faculty of Professional Studies, School of Kinesiology, Acadia University, Wolfville, NS, Canada

**Keywords:** aerobic fitness, endothelial function, prolonged sitting, resistance exercise, vasoconstrictor function

## Abstract

The cardiovascular benefits of physical exercise are well established. The vasoreactivity that occurs during reductions in local arterial blood flow, termed low-flow-mediated constriction (L-FMC), is a measure of endothelial-dependent vasoconstrictor function. It is unclear whether aerobic fitness and movement (or lack thereof) influences L-FMC. We systematically reviewed studies examining the impact of physical behaviours on L-FMC. To be included, cross-sectional and interventional studies had to examine the impact of a physical behaviour on L-FMC in adults. There were no language or date of publication restrictions. Sources were searched in May, 2021 and included Scopus, Embase, MEDLINE, CINAHL, and Academic Search Premier. National Institutes of Health quality assessment tools were used. Fourteen studies (15 arms; 313 participants; 398 total observations from four arteries) met the inclusion criteria. The study quality varied from four out of 14 (controlled intervention scoring) to nine out of 12 (longitudinal intervention with no control group scoring) with the total points dependent upon the study design. Conflicting results were reported for acute prolonged sitting studies (attenuated L-FMC: *n* = 1; no change: *n* = 1) and resistance exercise (increased L-FMC: *n* = 2; no change: *n* = 2). Most observational studies examining aerobic fitness (3/4 studies) and aerobic exercise interventions (4/5 studies) observed a favourable effect on L-FMC. Overall, the included studies support that higher aerobic fitness and engaging in aerobic exercise training may augment L-FMC responses. Our systematic review highlights the heterogeneity between studies and identifies current gaps and future directions to better our understanding of (in)activity, exercise, and posture on endothelial vasoconstrictor function. PROSPERO Registration No.: CRD42021248241

## Introduction

The adverse health consequences of peripheral vascular diseases are well established,^1^ with vascular diseases initially characterized by dysfunction of the endothelial cells that form the innermost layer of arteries.^2^ Endothelial cells are major contributors to ensuring proper vascular tone by balancing the release of vasodilator versus vasoconstrictor substances. Endothelial-dependent vasodilator function is commonly determined via the ultrasound-based flow-mediated dilation (FMD) technique.^3^ The FMD technique measures the increase in arterial lumen diameter in response to a reactive hyperemia elicited by a prior period of distal ischemia and provides an index of nitric oxide bioavailability.^4^ In addition to providing a measure of endothelial vasodilator function, the decline in conduit artery diameter observed during the distal ischemic period of the FMD test, termed low-flow-mediated constriction (L-FMC), provides an index of endothelial-dependent *vasoconstrictor* function.^5,6^ Although the L-FMC result is an underreported aspect of the ischemia-reactivity protocol in comparison to the commonly measured FMD, it provides unique information regarding endothelial function. The L-FMC response is unaffected by endothelial nitric oxide synthase blockade,^4^ but is attenuated when the production of endothelial-derived hyperpolarizing factors^7^ and prostaglandins^8^ are inhibited. Furthermore, blunted L-FMC responses have been observed following endothelin-1A receptor antagonism.^9^ A larger (i.e., more negative) L-FMC is a healthier endothelial response,^10^ with older adults^10^ and patients with coronary artery disease^8^ exhibiting attenuated L-FMC responses. A greater understanding of factors that influence this relatively understudied measure of vasoreactivity is warranted.

There are well-established cardiovascular health benefits of engaging in regular physical activity (e.g., high step counts) and having a greater cardiorespiratory fitness (i.e., maximal rate of oxygen consumption; V̇O_2max_).^11^ International organizations have incorporated whole-day approaches to their movement guidelines,^12^ including intensity-related aerobic physical activity, sedentary time (i.e., time spent sitting/reclining/lying), and resistance exercise. The favourable impact of exercise training interventions on FMD responses have been systematically reviewed elsewhere,^13,14^ but our understanding regarding the impact of physical behaviours on L-FMC is unclear. Importantly, L-FMC is a unique measure of endothelial function, with the known impact of movement on FMD not able to be extrapolated to L-FMC. Aerobic and resistance exercise training interventions test the impact of incorporating ~10–60 minutes of physical activity on training days, but it is important to also consider other habitual movement-focused factors that comprise activity-related guidelines, such as moderate–vigorous aerobic physical activity and sedentary time. Systematic reviews that examine the impact of physical lifestyle factors on vascular function should consider both aerobic fitness and the behaviours that encompass international guidelines.^12^

Whereas cross-sectional studies provide useful insight regarding habitual activities, between-group study designs cannot establish causal inference. Conversely, acute (i.e., immediate responses to single session) and longitudinal intervention studies implementing a repeated-measures design are a superior design for establishing causality. Accordingly, the objective of this study was to systematically review both cross-sectional and interventional studies that examine the impact of physical fitness or physical behaviours on L-FMC.

## Methods

### Search strategy

The search strategy and systematic review procedures were preregistered in PROSPERO (**Registration No.: CRD42021248241**). This review followed the preferred reporting for items for systematic reviews and meta-analysis (PRISMA) 2020 statement. Literature searches were conducted using Scopus, Embase, MEDLINE, CINAHL, and Academic Search Premier databases on May 16, 2021. Search strategies and results are presented in online Supplemental Table 1. Article citations were downloaded to an online research management system (EndNote, Clarivate Analytics, Philadelphia, PA, USA) and duplicates were removed. The remaining references were exported to a systematic review software for screening (Covidence, Melbourne, Australia).

### Study inclusion and exclusion criteria

Studies not published in a peer reviewed journal, or published as an editorial, review, opinions, or conference abstract were excluded. Grey literature would not provide sufficient information about the methodological procedures and outcomes to be fully included in the study and therefore were excluded. No language or timeline restrictions were applied. All studies included human adult participants (> 18 years). Studies were excluded if they did not include a measure of L-FMC (i.e., measure of vasoreactivity to a cuff-induced reduction in local blood flow).

Both observational and intervention studies were included. For cross-sectional studies, an objective measure of aerobic fitness (i.e., V̇O_2max_), physical activity (e.g., step counts, energy expenditure), physical activity intensity (e.g., moderate–vigorous physical activity), or sedentary time (e.g., sitting/lying) was required. Subjective measures were excluded. For cross-sectional studies, comparisons were made between groups or in the pooled sample (if applicable). Intervention studies must have included an experimental manipulation of physical behaviour (e.g., exercise training intervention, acute bout of sitting, etc.). For interventional studies, the comparator must have been a time-matched control group or alternative exercise intensity group. However, a comparator group was not required to be included in the review (e.g., single exercise training group with repeated measures). Both acute interventions (i.e., single session) and longitudinal intervention (i.e., multiple session) studies were included. No limit was placed on the duration of interventions.

### Study screening process

Two reviewers (JLP & YW) screened the titles and abstracts of all citations produced from the search. Next, the full text of apparently relevant articles was obtained and screened by the same two reviewers. If a consensus could not be reached, a third reviewer (MWO) was consulted as a tiebreaker. The reference list of included articles was hand-searched for other potentially relevant manuscripts.

### Data extraction

For included papers, participant characteristics (age, proportion of females, body mass index, health status), sample sizes, experimental design, intervention (if applicable; frequently, intensity, type, time), resting artery lumen diameter and local blood flow, as well as methodological considerations for the FMD and L-FMC protocol (e.g., artery of interest, cuff inflation pressure, etc.) were extracted. Our primary measures were: (1) the cross-sectional comparison between a measure of aerobic fitness or physical behaviour with L-FMC; (2) between-group comparisons of L-FMC based on differences in aerobic fitness or physical behaviour; or (3) L-FMC measured before and after an exercise, physical activity, or sedentary time intervention.

### Study quality assessment

The National Institutes of Health – National Heart, Lungs, and Blood Institute (NHLBI) quality assessment tools were used for each study design: controlled intervention studies, observational cohort and cross-sectional studies, and pre-post studies with no control group. The scoring of each tool is dependent upon the study design and is out of 12 or 14. The specific tool checklist and information on how to score each tool is presented on the NHLBI website (https://www.nhlbi.nih.gov/health-topics/study-quality-assessment-tools).

As with the article screening process, quality assessment was independently completed by two reviewers (JLP & YW). Reviewers met to discuss inconsistencies regarding their quality assessment decisions and if a resolution could not be agreed upon a senior third reviewer (NWB) was consulted to make a final decision.

## Results

**Figure 1.**
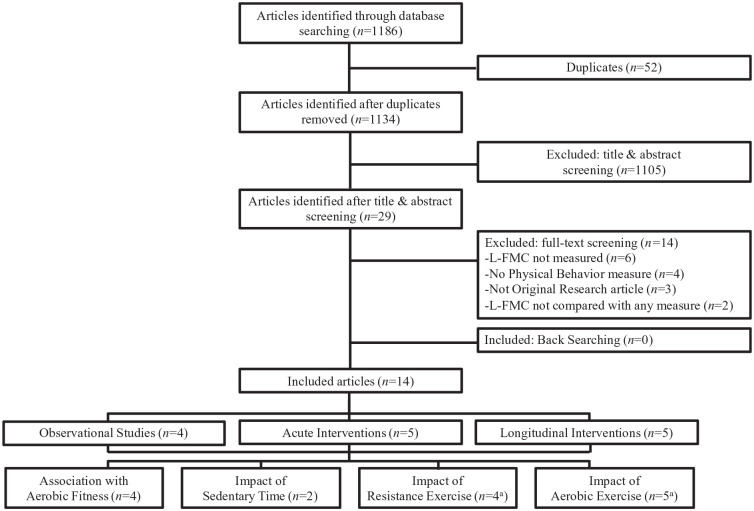
Flow chart indicating the number of articles included or excluded at each part of the screening process and breakdown of articles that are observational, acute interventions or longitudinal interventions. ^a^O’Brien et al. (2020)^23^ resistance training and aerobic training intervention arms were presented separately. L-FMC, low-flow-mediated constriction.

As shown in Figure 1, the systematic review produced 14 articles that met inclusion criteria, inclusive of cross-sectional (*n* = 4), acute intervention (*n* = 5), and longitudinal studies (*n* = 5). Of these, most were conducted in younger adults (*n* = 9; average: < 40 years), with fewer in middle-aged to older adults (*n* = 5; average > 50 years). Most studies (*n* = 11) were conducted in healthy populations, with some in patients requiring transradial catheterization (*n* = 1), obesity (*n* = 1), or chronic kidney disease (*n* = 1). Studies incorporated aerobic exercise interventions (*n* = 5), cross-sectional comparisons of aerobic fitness (*n* = 4), resistance exercise interventions (*n* = 3), or acute prolonged sitting interventions (*n* = 2). The 14 included studies incorporated a total of 398 observations (233 brachial, 105 popliteal, 40 radial, and 20 posterior tibial arteries) from 313 total participants (*n* = 163 females; 52% females). The study quality varied from four of 14 to nine of 12 (denominator total points dependent upon study design), with most of moderate quality as presented in online Supplemental Tables 2–4. Additional study characteristics, methodological details, and secondary results are presented in online Supplemental Table 5.

### Relationship with aerobic fitness

All cross-sectional studies (total: *n* = 157) examined the association between aerobic fitness (V̇O_2_peak) and L-FMC, with most (*n* = 3) focusing on the brachial artery^15–17^ and one on the popliteal.^18^ As presented in Table 1, three studies observed a moderate–strong negative correlation (i.e., healthier relationship; *R* larger than −0.5) between aerobic fitness and L-FMC in younger males^15^ and older adults (both males and females).^16,18^ However, Augustine et al.^17^ observed similar brachial artery L-FMC responses between amenorrheic female athletes, eumenorrheic female athletes, and a less aerobically fit control group. The study qualities ranged from five to eight (out of 14), with the null effect observed in the study^17^ with the lowest study quality (5/14).

**Table 1.** Included cross-sectional studies comparing measures of aerobic fitness with L-FMC.

Study	*n* (% female)	Age in years (BMI; kg/m^2^)	Aerobic fitness measure	Artery	Preocclusion diameter	L-FMC (%)	Summary
**Augustine et al.** ^17^	AA: 10 (100%) EA: 18 (100%) CON: 15 (100%)	AA: 21 ± 3 (22 ± 2) EA: 22 ± 3 (23 ± 2) CON: 23 ± 4 (24 ± 3)	V̇ O_2max_	Brachial	=	AA: −1.8 ± 4.3 EA: −1.6 ± 4.6 CON: −1.5 ± 2.8	=
**Bell et al.** ^15^	HF: 10 (0%) LF: 10 (0%)	HF: 22 ± 2 (24 ± 1) LF: 24 ± 6 (23 ± 4)	Estimated V̇ O_2max_	Brachial	=	HF: −2.6 ± 1.6 LF: −0.7 ± 1.8^a^ (*R* = −0.50^b^)	↑
**O’Brien et al.** ^16^	HF: 20 (55%) LF: 27 (70%)	HF: 66 ± 4 (25 ± 3) LF: 68 ± 5 (28 ± 5)	V̇ O_2max_	Brachial	HF↑	HF: −1.2 ± 0.9 LF: −0.5 ± 0.6^a^ (*R* = −0.52^b^)	↑
**O’Brien et al.** ^18^	47 (64%)	67 ± 5 (27 ± 4)	V̇ O_2max_	Popliteal	↑	–0.9 ± 1.4 (*R* = −0.73^b^)	↑

Data presented as means ± SDs or proportional (%).

Detailed information is presented as online Supplemental Table 5.

Arrows indicate the change from pre- to post-intervention; = indicates no difference or unrelated.

a Between-group statistical difference (*p* < 0.05); ^b^statistically significant correlation (*R*, Pearson correlation).

AA, amenorrheic athletes; BMI, body mass index; CON, control group; EA, eumenorrheic athletes; HF, higher aerobic fitness; LF, lower aerobic fitness; L-FMC, low-flow-mediated constriction; V̇ O_2max_, maximal rate of oxygen consumption.

### Impact of acute prolonged sitting

Two studies (total: *n* = 40) examined the impact of prolonged sitting (Table 2) on L-FMC. Both were conducted in young, healthy adults with each measurement conducted immediately after a 3-hour sitting protocol. L-FMC was unchanged in the posterior tibial artery^19^ but attenuated in the popliteal artery.^20^ Both studies were of a similar moderate quality (both, 6/12).

**Table 2.** Included studies examining the impact of sedentary postures on L-FMC.

Study	*n* (% female)	Age in years (BMI; kg/m^2^)	Intervention	Artery	Preocclusion diameter	L-FMC (%)	Summary
**Credeur et al.** ^19^	20 (35%)	26 ± 7 (30 ± 7)	3-h bout of prolonged sitting	Posterior tibial	=	*Absolute* Pre: −0.4 ± 0.5 mm Post: −0.4 ± 0.5 mm Estimated relative Pre: −17.0 Post: −20.5	=
**O’Brien et al.** ^20^	20 (50%)	G1: 24 ± 2 (27 ± 2) G2: 23 ± 2 (24 ± 3)	3-h bout of prolonged sitting in males (G1) and females (G2)	Popliteal	G1: = G2: =	*G1* Pre: −1.7 ± 1.0 Post: −0.4 ± 0.5^a^ G2 Pre: −1.9 ± 0.9 Post: −0.5 ± 0.6^a^	G1: ↓ G2: ↓

Data presented as means ± SDs or proportional (%).

Detailed information is presented as online Supplemental Table 5.

Arrows indicate the change from pre- to post-intervention; = indicates no difference or unrelated.

a Pre-post statistical difference (*p* < 0.05).

BMI, body mass index; G1, group 1; G2, group 2; L-FMC, low-flow-mediated constriction; ↓, a smaller, less negative L-FMC.

### Impact of resistance exercise interventions

Two acute and two longitudinal (6-week) intervention studies (total: *n* = 51) examined the impact of resistance exercise on L-FMC (two brachial, two radial, one popliteal; Table 3). Choi et al.^21^ observed no statistical change in the nonexercising limb (i.e., brachial L-FMC) among their small sample (*n* = 7) of young males following 50 maximal effort eccentric contralateral elbow flexion contractions. Conversely, Gori et al.^22^ observed augmented radial L-FMC following 4 minutes of rhythmic ipsilateral handgrip exercise compared to the time-controlled condition in a group of young adults. Choi et al.^21^ and Gori et al.^22^ had overall study quality ratings of five out of 12 and four out of 14, respectively.

**Table 3.** Included studies examining the impact of resistance exercise on L-FMC.

Study	*n* (% female)	Age in years (BMI; kg/m^2^)	Intervention	Artery	Preocclusion diameter	L-FMC (%)	Summary
***Acute interventions***							
**Choi et al.** ^21^	7 (0%)	24 ± 3 (22 ± 2)	Single bout of 50 eccentric isokinetic arm contractions	Brachial	=	Pre: −2.6 ± 2.4 Post: −4.1 ± 2.9	=
***Longitudinal interventions***							
**Gori et al.** ^22^	12 (42%)	Range: 25–35 (NR)	I1: Single bout of 4 min rhythmic handgrip I2: Time control	Radial	I1: ↑ I2: =	I1: Post: −5.1 ± 1.3 I2: Post: −7.9 ± 3.3^a^	↑
**Dawson et al.** ^5^	I1: 9 (11%) I2: 9 (44%)	I1: 63 ± 7 (27) I2: 66 ± 9 (27)	I1: 6 weeks of moderate-intensity handgrip exercise I2: Time control	Radial	I1: = I2: =	I1 Pre: −2.1 ± 4.3 Post: −3.6 ± 3.1 I2 Pre: −3.3 ± 3.6 Post: −1.3 ± 3.8^b^	I1: = I2: ↓
**O’Brien et al.** ^23^	14 (57%)	66 ± 7 (27 ± 5)	6 weeks of whole-body resistance training	Brachial	↑	Pre: −0.8 ± 0.6 Post: −0.9 ± 0.7	=
				Popliteal	↑	Pre: −1.0 ± 1.7 Post: −1.0 ± 1.1	=

Data presented as means ± SDs or proportional (%).

Detailed information is presented as online Supplemental Table 5.

The O’Brien et al. (2020)^23^ study was broken into resistance training and aerobic training components.

Arrows indicate the change from pre- to post-intervention; = indicates no difference or unrelated.

a Statistical difference between postintervention time points; ^b^pre-post statistical difference (*p* < 0.05).

BMI, body mass index; I1, first intervention group; I2, second intervention group; L-FMC, low-flow-mediated constriction; NR, not reported; ↑, a larger more negative L-FMC; ↓, a smaller less negative L-FMC.

In older adults requiring trans-radial catherization for coronary angiography or angioplasty, Dawson et al.^5^ (quality: 4/14) observed that 6 weeks of bilateral moderate-intensity handgrip exercise (30 min, 30 contractions/min at 40% maximum voluntary contraction, three times/week) preserved radial L-FMC, whereas the nonexercising control group exhibited an attenuated L-FMC response (Table 3). In healthy older adults, O’Brien et al.^23^ (quality: 9/12) observed that 6 weeks of whole-body resistance training did not alter brachial or popliteal L-FMC, despite increasing the preocclusion diameter in both arteries.

### Impact of aerobic exercise interventions

One acute and four longitudinal intervention studies (total *n* = 112) examined the impact of aerobic exercise on L-FMC (four brachial, one radial, one popliteal; Table 4). In a sample of young males, Elliott et al.^24^ (quality: 7/14) demonstrated that 30 minutes of low to moderate-intensity cycling (50–150 W) increased radial L-FMC but demonstrated no change following a nonexercise time control.

**Table 4.** Included studies examining the impact of aerobic exercise on L-FMC.

Study	*n* (% female)	Age in years (BMI; kg/m^2^)	Intervention	Artery	Preocclusion diameter	L-FMC (%)	Summary
***Acute interventions***							
**Elliott et al.** ^24^	10 (0%)	23 ± 4 (24)	I1: Single 30-min bout of low/moderate-intensity cycling I2: Time control	Radial	I1: ↑ I2: ↓	I1: Pre: −5.6 ± 3.3 Post: −10.1 ± 3.8^a^ I2: Pre: −8.1 ± 3.2 Post: −6.7 ± 3.4	I1: ↑ I2: =
***Longitudinal interventions***							
**O’Brien et al.** ^23^	I1: 12 (67%) I2: 12 (58%)	I1: 68 ± 6 (25 ± 4) I2: 68 ± 5 (26 ± 3)	I1: 6 weeks of moderate-intensity continuous training I2: High-intensity interval training	Brachial	I1: = I2: =	I1: Pre: 0.9 ± 1.0 Post: −1.2 ± 0.9 I2: Pre: −0.9 ± 1.7 Post: −1.7 ± 1.2^a^	I1: = I2: =
				Popliteal	I1: = I2: =	I1: Pre: −0.8 ± 1.6 Post: −1.5 ± 0.7^a^ I2: Pre: −0.9 ± 1.1 Post: −1.8 ± 0.9^a^	I1: ↑ I2: ↑
**Rakobowchuk et al.** ^25^	I1: 9 (67%) I2: 11 (55%)	I1: 24 ± 3 (25 ± 2) I2: 23 ± 3 (23 ± 3)	I1: 6 weeks of moderate-intensity interval cycling I2: Heavy-intensity interval cycling	Brachial	I1: = I2: =	I1: Pre: −0.5 ± 3.2 Post: −1.9 ± 3.1^a^ I2: Pre: −1.1 ± 1.6 Post: −3.0 ± 3.0^a^	I1: ↑ I2: ↑
**Sawyer et al.** ^26^	I1: 9 (55%) I2: 9 (45%)	I1: 35 ± 8 (35 ± 3) I2: 36 ± 9 (37 ± 6)	I1: 8 weeks of moderate-intensity cycling I2: High-intensity interval cycling	Brachial	I1: ↑ I2: =	I1: Pre: 0.6 ± 2.0 Post: −2.8 ± 3.2^a^ I2: Pre: −1.0 ± 4.1 Post: 1.7 ± 3.5	I1: ↑ I2: =
**Van Craenenbroeck et al.** ^27^	I1: 19 (42%) I2: 21 (48%)	I1: 52 ± 12 (28 ± 6) I2: 55 ± 14 (28 ± 6)	I1: 12 weeks of moderate-intensity cycling I2: Usual care	Brachial	I1: = I2: =	I1: Pre: −0.3 ± 2.2 Post: −0.1 ± 2.1 I2: Pre: −0.9 ± 1.6 Post: −0.8 ± 2.8	I1: = I2: =

Data presented as means ± SDs or proportional (%).

Detailed information is presented as online Supplemental Table 5.

The O’Brien et al. (2020)^23^ study was broken into resistance training and aerobic training components.

Arrows indicate the change from pre- to post-intervention; = indicates no difference or unrelated.

a Pre-post statistical difference (*p* < 0.05).

BMI, body mass index; I1, first intervention group; I2, second intervention group; L-FMC, low-flow-mediated constriction; ↑, a larger more negative L-FMC; ↓, a smaller, less negative L-FMC.

In younger adults, Rakobowchuk et al.^25^ (quality: 5/12) demonstrated increases in brachial L-FMC following 6 weeks of moderate-intensity or heavy-intensity interval cycling. In contrast, Sawyer et al.^26^ (quality: 8/12) did not observe changes in brachial L-FMC in young, obese adults following 8 weeks of high-intensity interval cycling, but moderate-intensity cycling augmented brachial L-FMC (see online Supplemental Table 5 for exercise program details).

In middle-aged to older adults with stage 3 or 4 chronic kidney disease, Van Craenenbroeck et al.^27^ (quality: 9/14) did not observe any changes in brachial L-FMC in response to 12 weeks of either home-based cycling (four bouts × 10 min daily at 90% maximum heart rate) or usual care. In healthy older adults (quality: 9/12), 6 weeks of moderate-intensity continuous cycling (34 min at 60% peak power output) and high-intensity interval cycling (40 min of 15 s:15 s at 100% peak power output:passive recovery) both improved popliteal L-FMC, but only the interval training augmented brachial L-FMC.^23^

## Discussion

The purpose of this study was to systematically review observational and interventional studies examining the impact of physical behaviours (i.e., sedentary time, aerobic fitness, physical exercise interventions) on L-FMC. Overall, 14 articles were included, with the majority (10/14) observing an effect of physical behaviours to alter endothelial-dependent vasoconstriction. Overall, these results generally support that higher aerobic fitness and engaging in aerobic exercise is associated with better endothelial sensitivity to low flow.

Three of four studies demonstrated that higher aerobic fitness was associated with a larger L-FMC response in the brachial artery of young males,^15^ brachial artery of healthy older adults,^16^ and popliteal artery of healthy older adults.^18^ In contrast, brachial L-FMC was similar between amenorrheic/eumenorrheic female athletes (V̇O_2max_: ~50 mL/kg/min) and less aerobically fit eumenorrheic controls (V̇O_2max_: ~38 mL/kg/min).^17^ Whether the relationship between aerobic fitness and L-FMC is sex-specific warrants further research. Unlike FMD, L-FMC is primarily mediated via the inhibition of endothelial-derived hyperpolarizing factor^7^ and prostaglandins,^8^ as well as enhanced vasoconstrictor signalling via endothelin-1.^9^ In support of the inverse relationship between L-FMC and aerobic fitness, basal endothelin-1 levels are typically lower in older adults who are more aerobically fit^28^ and exercise training improves the signalling of endothelial-derived hyperpolarizing factor^29^ and prostaglandins^30^ in rodent models. Future mechanistic studies are warranted to determine which of these specific individual or combinations of factors are involved with the greater L-FMC responses observed in more aerobically fit adults. Although it is presumed that the beneficial effects of aerobic fitness level on L-FMC are movement-related, it cannot be directly confirmed whether genetic or diet-related factors (e.g., sodium intake^31^) influenced these cross-sectional observations.

International activity recommendations promote the inclusion of two resistance training sessions per week.^12^ A previous systematic review has demonstrated that resistance training improves FMD responses, albeit to a lesser extent than aerobic training.^13^ This study is the first to systematically review the impact of resistance exercise on L-FMC, and demonstrates conflicting reports based on four studies of low–moderate quality. The two studies to conduct handgrip exercise demonstrated an increase of L-FMC to acute exercise^22^ and a preserved L-FMC in response to a handgrip intervention versus the control group.^5^ The null observations observed in other studies may be due to the nonlocalized cardiovascular responses to whole-body resistance training^23^ or the measurement of L-FMC in the nonexercise arm.^21^ Accordingly, the divergent findings may be attributed to the localized effects of handgrip training on upper-limb vasoconstrictor function. Alternatively, the two studies that observed an effect were conducted in the radial artery, which may exhibit a more consistent L-FMC response than the brachial artery.^32^

Acutely, Elliott et al.^24^ demonstrated that low/moderate-intensity cycling augmented radial L-FMC. Although the acute FMD responses to a single bout of exercise may be predictive of the vascular adaptation to chronic exercise training,^33^ it is unclear if a similar response exists for L-FMC. Of the interventional studies included, three of the four studies observed a favourable impact of aerobic exercise training on L-FMC. The three studies that observed a positive effect consisted of supervised training sessions. In contrast, the study by Van Craenenbroeck et al.^27^ consisted of multiple (four times/day), 10-min sessions of cycling at home in patients with stage 3 or 4 chronic kidney disease. As such, it is plausible that differences in the supervision of participants (i.e., attendance of sessions), exercise duration, or health status of the participants are responsible for the divergent results.

The included studies examined L-FMC responses in the brachial, radial, popliteal, or posterior tibial arteries. The magnitude of L-FMC responses vary between the brachial artery versus the popliteal^18^ or radial^32^ arteries. Upper-limb FMD responses may provide some clinically relevant information regarding coronary artery function,^34^ but a similar clinical value is not established for L-FMC responses. Lower-limb vessels are more susceptible to the development of atherosclerosis^35^ and are subjected to larger oscillations in local blood flow patterns during bouts of physical activity and sedentary postures. Therefore, it seems logical to study lower-limb arteries when probing the endothelial responses to aerobic fitness or movement (or lack thereof). The results of our review identify conflicting reports regarding the impact of acute prolonged sitting time on L-FMC. Given that sitting reduces leg blood flow and is the posture that comprises the majority of our waking hours,^36^ the impact of sedentary behaviours on the endothelial sensitivity to low flow may provide valuable information. Examining L-FMC responses in the lower-limb vasculature in response to sedentary behaviour interventions are needed to confirm or tweak public health guidelines that recommend reducing sedentary time and frequently breaking up sitting.^12^ Based on the gaps in the literature identified in this review, we have provided some recommendations and future directions that may improve our understanding of L-FMC and the potential impact of aerobic fitness and physical behaviours (Table 5).

**Table 5.** Recommendations for future L-FMC research.

• Impact of objectively measured habitual physical activity, sedentary time, and sedentary patterns on L-FMC.
• The independent influence of aerobic fitness versus physical activity on L-FMC.
• High-quality randomized control trials on the impact of exercise type (aerobic, resistance, etc.) and intensity (moderate/high-intensity interval, etc.) on L-FMC.
• Impact of ischemic resistance exercise and handgrip duty cycle on L-FMC.
• Impact of movement on L-FMC in patients with cardiovascular conditions (i.e., cardiac rehabilitation, peripheral artery disease, etc.).
• Impact of longer (> 12 weeks) exercise, activity, or sedentary time interventions on L-FMC.
• Mechanisms (endothelial-derived hyperpolarizing factor, prostaglandins, and endothelin-1) responsible for movement-related increase in L-FMC.
• Prognostic value of L-FMC in different arteries and if movement-related improvements in L-FMC provide clinically relevant information.
• Inclusion of lower-limb vessels that are directly involved in supplying muscle mass with blood flow during physical behaviours.
• Determine the impact of L-FMC measurement period (average 30s or nadir) and reach a consensus on best time to use.
• Investigate normalization procedures for L-FMC and if the same procedures for FMD should be used (reactive hypoemia, allometric scaling, etc.).

FMD, flow-mediated dilation; L-FMC, low-flow-mediated constriction.

As outlined in our preregistered study design (PROSPERO Registration No.: CRD42021248241), there was an intention of conducting a meta-analysis if data were sufficiently homogenous. However, the heterogeneous nature of the domains studied (e.g., aerobic fitness, sedentary time, etc.), interventions implemented, and arteries examined prevented the amalgamations of studies for a meta-analysis. Our review may be limited by the number of studies conducted and the quality of studies, which were generally of moderate quality. Nevertheless, the outcomes of this review support that higher aerobic fitness and exercise training can augment L-FMC and provides direction for future cross-sectional and interventional studies in the field. Given most included articles were published in the last 5 years (nine out of 14), this is a timely review of a relatively understudied but important measure of vascular function and will direct further study in this field.

## Conclusion

Our systematic review highlights the heterogeneity between studies and identifies current gaps and future directions to better our understanding of (in)activity, exercise, and posture on vasoconstrictor function. Overall, the included studies demonstrated that higher aerobic fitness and engaging in aerobic exercise training augmented L-FMC responses. Future observational and interventional studies are needed to better characterize the influence of sedentary postures, habitual physical activity, and resistance training.

## Supplemental Material

sj-pdf-1-vmj-10.1177_1358863X211073480 – Supplemental material for What is the impact of aerobic fitness and movement interventions on low-flow-mediated vasoconstriction? A systematic review of observational and intervention studies Supplemental material, sj-pdf-1-vmj-10.1177_1358863X211073480 for What is the impact of aerobic fitness and movement interventions on low-flow-mediated vasoconstriction? A systematic review of observational and intervention studies by Myles W O’Brien, Jennifer L Petterson, Yanlin Wu, Nick W Bray and Derek S Kimmerly in Vascular Medicine

sj-pdf-2-vmj-10.1177_1358863X211073480 – Supplemental material for What is the impact of aerobic fitness and movement interventions on low-flow-mediated vasoconstriction? A systematic review of observational and intervention studies Supplemental material, sj-pdf-2-vmj-10.1177_1358863X211073480 for What is the impact of aerobic fitness and movement interventions on low-flow-mediated vasoconstriction? A systematic review of observational and intervention studies by Myles W O’Brien, Jennifer L Petterson, Yanlin Wu, Nick W Bray and Derek S Kimmerly in Vascular Medicine

sj-pdf-3-vmj-10.1177_1358863X211073480 – Supplemental material for What is the impact of aerobic fitness and movement interventions on low-flow-mediated vasoconstriction? A systematic review of observational and intervention studies Supplemental material, sj-pdf-3-vmj-10.1177_1358863X211073480 for What is the impact of aerobic fitness and movement interventions on low-flow-mediated vasoconstriction? A systematic review of observational and intervention studies by Myles W O’Brien, Jennifer L Petterson, Yanlin Wu, Nick W Bray and Derek S Kimmerly in Vascular Medicine

sj-pdf-4-vmj-10.1177_1358863X211073480 – Supplemental material for What is the impact of aerobic fitness and movement interventions on low-flow-mediated vasoconstriction? A systematic review of observational and intervention studies Supplemental material, sj-pdf-4-vmj-10.1177_1358863X211073480 for What is the impact of aerobic fitness and movement interventions on low-flow-mediated vasoconstriction? A systematic review of observational and intervention studies by Myles W O’Brien, Jennifer L Petterson, Yanlin Wu, Nick W Bray and Derek S Kimmerly in Vascular Medicine

sj-pdf-5-vmj-10.1177_1358863X211073480 – Supplemental material for What is the impact of aerobic fitness and movement interventions on low-flow-mediated vasoconstriction? A systematic review of observational and intervention studies Supplemental material, sj-pdf-5-vmj-10.1177_1358863X211073480 for What is the impact of aerobic fitness and movement interventions on low-flow-mediated vasoconstriction? A systematic review of observational and intervention studies by Myles W O’Brien, Jennifer L Petterson, Yanlin Wu, Nick W Bray and Derek S Kimmerly in Vascular Medicine
