# Supplementary material for: What is the impact of aerobic fitness and movement interventions on low-flow-mediated vasoconstriction? A systematic review of observational and intervention studies
Source: Vasc Med. 2022 Feb 24;27(2):193–202. doi: 10.1177/1358863X211073480 (PMC11909780; doi:10.1177/1358863X211073480)
Supplement: sj-pdf-1-vmj-10.1177_1358863X211073480 – Supplemental material for What is the impact of aerobic fitness and movement interventions on low-flow-mediated vasoconstriction? A systematic review of observational and intervention studies [file sj-pdf-1-vmj-10.1177_1358863X211073480.pdf]

Supplemental Table 1. Databases searched and the search strategy.

**Database (number of results)**
MEDLINE (*n*=1014)
Scopus (*n*=109)
EMBASE (*n*=10)
Academic Search Premier (*n*=45)
CINAHL (*n*=8)
Total (*n*=1186)
**Search Strategy**
1. “Low-flow mediated constriction”
2. “L-FMC”
3. “LFMC”
4. “Endothelial-dependent vasoconstriction”
5. “Shear-mediated vasoconstriction”
6. “Conduit artery constriction”
7. “Resting endothelial function”
8. 1 OR 2 OR 3 OR 4 OR 5 OR 6 OR 7
9. “cerebral”
10. “Aerobic fitness”
11. “Cardiorespiratory fitness”
12. “Cardiovascular fitness”
13. “Maximal aerobic uptake”
14. “Peak aerobic uptake”
15. “Aerobic exercise”
16. “Resistance exercise”
17. “Physical exercise”
18. “High-intensity exercise”
19. “Exercise training”
20. “Aerobic training”
21. “Physical activity”
22. “Step counts”
23. “Moderate-vigorous”
24. “Sedentary”
25. “Sitting”
26. 10 OR 11 OR 12 OR 13 OR 14 OR 15 OR 16 OR 17 OR 18 OR 19 OR 20 OR 21 OR 22 OR 23 OR 24 OR 25
27. 8 AND 26 NOT 9

Note: The search strategy was adapted for each database by using brackets, backslashes or quotations around terms as needed.
