# Supplementary material for: What is the impact of aerobic fitness and movement interventions on low-flow-mediated vasoconstriction? A systematic review of observational and intervention studies
Source: Vasc Med. 2022 Feb 24;27(2):193–202. doi: 10.1177/1358863X211073480 (PMC11909780; doi:10.1177/1358863X211073480)
Supplement: sj-pdf-3-vmj-10.1177_1358863X211073480 – Supplemental material for What is the impact of aerobic fitness and movement interventions on low-flow-mediated vasoconstriction? A systematic review of observational and intervention studies [file sj-pdf-3-vmj-10.1177_1358863X211073480.pdf]

Supplemental Table 3. Study quality assessment for pre-post studies with no control group

**Quality Question**	**Choi et al. (2016)**	**Credeur et al. (2019)**	**O'Brien et al. (2020)**	**O'Brien et al. (2019c)**	**Rakowbo chuk et al. (2012)**	**Sawyer et al. (2012)**
1. Was the study question or objective clearly stated?	1	1	1	1	1	1
2. Were eligibility/selection criteria for the study population prespecified and clearly described?	1	1	1	1	1	1
3. Were the participants in the study representative of those who would be eligible for the test/service/intervention in the general or clinical population of interest?	0	0	1	0	0	1
4. Were all eligible participants that met the prespecified entry criteria enrolled?	NR	NR	NR	NR	0	NR
5. Was the sample size sufficiently large to provide confidence in the findings?	0	1	0	0	0	1
6. Was the test/service/intervention clearly described and delivered consistently across the study population?	1	1	1	1	1	1
7. Were the outcome measures prespecified, clearly defined, valid, reliable, and assessed consistently across all study participants?	1	1	1	1	1	1
8. Were the people assessing the outcomes blinded to the participants' exposures/interventions?	0	0	1	1	0	CD
9. Was the loss to follow-up after baseline 20% or less? Were those lost to follow-up accounted for in the analysis?	NA	NA	1	NA	0	1
10. Did the statistical methods examine changes in outcome measures from before to after the intervention? Were statistical tests done that provided p values for the pre-to-post changes?	1	1	1	1	1	1
11. Were outcome measures of interest taken multiple times before the intervention and multiple times after the intervention (i.e., did they use an interrupted time-series design)?	0	0	0	0	0	0
12. If the intervention was conducted at a group level (e.g., a whole hospital, a community, etc.) did the statistical analysis take into account the use of individual-level data to determine effects at the group level?	NA	NA	1	NA	NA	NA
**Total (out of 12)**	5	6	9	6	5	8

NR, not reported; CD, cannot determine; NA, not available.
