# Supplementary material for: What is the impact of aerobic fitness and movement interventions on low-flow-mediated vasoconstriction? A systematic review of observational and intervention studies
Source: Vasc Med. 2022 Feb 24;27(2):193–202. doi: 10.1177/1358863X211073480 (PMC11909780; doi:10.1177/1358863X211073480)
Supplement: sj-pdf-4-vmj-10.1177_1358863X211073480 – Supplemental material for What is the impact of aerobic fitness and movement interventions on low-flow-mediated vasoconstriction? A systematic review of observational and intervention studies [file sj-pdf-4-vmj-10.1177_1358863X211073480.pdf]

Supplemental Table 4. Study quality assessment for controlled intervention studies

**Quality Question**	**Dawson et al. (2012)**	**Elliott et al. (2018)**	**Gori et al. (2010)**	**Van Craenen broeck et al. (2015)**
1. Was the study described as randomized, a randomized trial, a randomized clinical trial, or an RCT?	1	1	1	1
2. Was the method of randomization adequate (i.e., use of randomly generated assignment)?	NR	1	NR	1
3. Was the treatment allocation concealed (so that assignments could not be predicted)?	NR	1	NR	1
4. Were study participants and providers blinded to treatment group assignment?	0	0	0	0
5. Were the people assessing the outcomes blinded to the participants' group assignments?	NR	1	1	1
6. Were the groups similar at baseline on important characteristics that could affect outcomes (e.g., demographics, risk factors, co-morbid conditions)?	1	1	1	1
7. Was the overall drop-out rate from the study at endpoint 20% or lower of the number allocated to treatment?	NR	NR	NR	0
8. Was the differential drop-out rate (between treatment groups) at endpoint 15 percentage points or lower?	NR	NR	NR	1
9. Was there high adherence to the intervention protocols for each treatment group?	NR	NR	NR	1
10. Were other interventions avoided or similar in the groups (e.g., similar background treatments)?	1	1	NR	1
11. Were outcomes assessed using valid and reliable measures, implemented consistently across all study participants?	1	1	1	0
12. Did the authors report that the sample size was sufficiently large to be able to detect a difference in the main outcome between groups with at least 80% power?	0	0	0	1
13. Were outcomes reported or subgroups analyzed prespecified (i.e., identified before analyses were conducted)?	0	0	0	CD
14. Were all randomized participants analyzed in the group to which they were originally assigned, i.e., did they use an intention-to-treat analysis?	NR	0	NR	0
**Total (out of 14)**	4	7	4	9

NR, not reported; CD, cannot determine.
