# Supplementary material for: What is the impact of aerobic fitness and movement interventions on low-flow-mediated vasoconstriction? A systematic review of observational and intervention studies
Source: Vasc Med. 2022 Feb 24;27(2):193–202. doi: 10.1177/1358863X211073480 (PMC11909780; doi:10.1177/1358863X211073480)
Supplement: sj-pdf-5-vmj-10.1177_1358863X211073480 – Supplemental material for What is the impact of aerobic fitness and movement interventions on low-flow-mediated vasoconstriction? A systematic review of observational and intervention studies [file sj-pdf-5-vmj-10.1177_1358863X211073480.pdf]

Supplemental Table 5. Additional characteristics and details of included studies.

Study	Sample	Intervention Length (# sessions)	Intervention	Occlusion pressure (Time)	Occlusion Location	L-FMC Measure	Pre-Occlusion Diameter	Pre-Occlusion Blood Flow	FMD (%)
Cross-sectional Studies									
**Augustine et al.**[19]	Healthy	-	-	200 mmHg (5 min)	Distal antecubital fossa	30s	AA: 3.2±0.3<br>EA: 3.2±0.4<br>CON: 3.0±0.3	NR	AA: 7.2±4.2<br>EA: 11.1±4.3<br>CON: 10.3±3.9
**Bell et al.**[17]	Healthy	-	-	220 mmHg (5 min)	Distal antecubital fossa	Nadir	HF: 4.1±0.5<br>LF: 3.9±0.4	NR	HF: 5.9±2.0<br>LF: 7.1±2.1
**O'Brien et al.**[18]	Healthy	-	-	250 mmHg (5 min)	Distal antecubital fossa	Nadir	HF: 4.2±0.8<br>LF: 3.8±0.5*	HF:104±40<br>LF: 80±37	HF: 5.7±1.5<br>LF: 3.9±0.6*
**O'Brien et al.**[20]	Healthy	-	-	250 mmHg (5 min)	Distal popliteal fossa	Nadir	6.6±1.4	88±48	3.3± 2.0
Acute Intervention Studies									
**Choi et al.**[22]	Healthy	1 session	1 set of 50 eccentric contractions isokinetic arm exercises	50 mmHg above SBP (5 min)	Distal antecubital fossa	10s post-deflation	Pre: 3.8±0.2<br>Post: 3.8±0.2	NR	Pre: 7.1± 0.7<br>Post: 4.3±0.8
**Credeur et al.**[21]	Healthy	1 session	3-hr uninterrupted sitting	220 mmHg (5 min)	Distal to fibular head	Nadir	Pre: 2.3±0.5<br>Post: 1.9±0.5	NR	Pre: 22±13<br>Post: 17±13
**Elliott et al.**[25]	Healthy	1 session	30 min bout of cycling (10min: 50W, 10 min: 100W, 10 min: 150 W)	240 mmHg (5 min)	Wrist	30s	I1: Pre: 2.6±0.2<br>Post: 2.7±0.3*<br>I2: Pre: 2.7±0.3<br>Post: 2.6±0.3	I1: Pre: 44±19<br>Post: 58±23*<br>I2: Pre: 51±25<br>Post: 27±19*	I1: Pre: 5.4±1.4<br>Post: 5.1±1.5<br>I2: Pre: 5.5±1.1<br>Post: 6.9±1.2
**Gori et al.**[23]	Healthy	1 session	4 mins rhythmic	Suprasystolic pressure (4.5 min)	Wrist	30s	I1: 2.3±0.3<br>I2: 2.3±0.3	I1: 10±12<br>I2: 8±7	I1: 3.1±3.2<br>I2: 6.0±2.3**

			handgrip contractions						
**O'Brien et al.**[16]	Healthy	1 session	3-hr uninterrupted sitting	250 mmHg<br>(5 min)	Distal popliteal fossa	Nadir	G1: Pre: 6.3±0.8<br>Post: 6.2±0.8<br>G2: Pre: 5.4±0.5<br>Post: 5.2±0.6	G1: Pre: 79±20<br>Post: 18±6*<br>G2: Pre: 55±10<br>Post: 12±4*	G1: Pre: 4.8±1.1<br>Post: 2.0±0.7*<br>G2: Pre: 4.5±0.7<br>Post: 1.9±0.7*
Longitudinal Intervention Studies									
**Dawson et al.**[5]	Patients requiring transradial catheterization	6-wk<br>(18 sessions)	30 contractions per min for 30 mins at 40% max	200 mmHg<br>(5 min)	Wrist	30s	I1: Pre: 2.9±0.3<br>Post: 2.8±0.5<br>I2: Pre: 2.6±0.5<br>Post: 2.7±0.3	I1: Pre: 50±35<br>Post: 38±27<br>I2: Pre: 32±20<br>Post: 33±24	I1: Pre: 6.8±2.4<br>Post: 6.9±3.5<br>I2: Pre: 8.3±4.6<br>Post: 4.7± 2.1
**O'Brien et al.**[24] **- Brachial**	Healthy	6-wk<br>(18 sessions)	I1: 34 mins at 60% PPO<br>I2: 15s:15s for 40 mins 100%PPO: 0%PPO<br>I3: 8 whole body resistance exercises	250 mmHg<br>(5 min)	Distal antecubital fossa	Nadir	I1: Pre: 3.9±0.5<br>Post: 3.9±0.5<br>I2: Pre: 4.3±0.7<br>Post: 4.3 ±0.7<br>I3: Pre: 3.7±0.6<br>Post: 3.7±0.6*	I1: Pre: 90±38<br>Post: 93±41<br>I2: Pre: 102±43<br>Post: 120 ±42*<br>I3: Pre: 83±36<br>Post: 78 ±28	I1: Pre: 4.7±1.9<br>Post: 6.8 ±1.7*<br>I2: Pre: 4.8±1.8<br>Post: 6.7±1.3*<br>I3: Pre: 4.7±1.4<br>Post: 5.0±1.4
**O'Brien et al.**[24] **- Popliteal**					Distal popliteal fossa	Nadir	I1: Pre: 6.7±2.0<br>Post: 6.6±2.0<br>I2: Pre: 6.4±1.1<br>Post: 6.4±1.1<br>I3: Pre: 6.9±0.9<br>Post: 7.0±0.8*	I1: Pre: 75±38;<br>Post: 83±37<br>I2: Pre: 111±54<br>Post: 110±56<br>I3: Pre: 96±55<br>Post: 95±47	I1: Pre: 2.6±1.7<br>Post: 4.0±1.9*<br>I2: Pre: 3.6±1.9<br>Post: 4.9±1.5*<br>I3: Pre: 3.1±1.9<br>Post: 3.4±1.8
**Rakobowchuk et al.**[26]	Healthy	6-wk<br>(18 sessions)	I1: 30-40 mins of 10s:20s interval of 120%PPO: 20W<br>I2: same but 30s:60s	NR<br>(5 min)	Distal to probe	Nadir	I1: Pre: 3.7±0.7<br>Post: 3.7±0.7<br>I2: Pre: 3.3±0.7<br>Post: 3.4±0.7	I1: Pre: 36±27<br>Post: 54±35*<br>I2: Pre: 41± 23<br>Post: 50±29*	I1: Pre: 5.4±3.5<br>Post: 6.6±2.6<br>I2: Pre: 6.7±4.1<br>Post: 7.1±3.4

**Sawyer et al.**[27]	Obese Adults	8-wk (24 sessions)	I1: 30 mins cycling at 70-75% HRmax I2: Ten 60s:60s intervals at 90-95% HRmax: 25-50W	250 mmHg (5 mins)	Forearm	Nadir	I1: Pre: 3.7±0.6 Post: 3.9±0.6* I2: Pre: 4.0±0.7 Post: 4.1±0.7	NR	I1: Pre: 6.0±4.1 Post: 3.2±3.1 I2: Pre: 4.8±2.7 Post: 8.6±5.3
**Van Craenenbroeck et al.**[28]	Patients with Chronic Kidney Disease	12-wk (4 unsupervised sessions per day)	10 mins at 90% HRmax	200 mmHg or 50+ SBP (5 min)	Forearm	30s	I1: Pre: 3.8±0.7 Post: 3.7±0.7 I2: Pre: 2.7±0.6 Post: 2.6±0.5	NR	I1: Pre: 4.0±1.9 Post: 4.6±3.0 I2: Pre: 5.2±3.4 Post: 5.3±3.1

Data presented as means ± standard deviations or proportional (%). FMD, flow-mediated dilation; L-FMC, low-flow-mediated constriction; HF, higher aerobic fitness; LF, lower aerobic fitness; BMI, body mass index; I1, first intervention group; I2, second intervention group; I3, third intervention group; G1, group 1; G2, group 2; EA, eumenorrheic athletes; CON, control group; NR, not reported; HRmax, maximum heart rate; SBP, systolic blood pressure; PPO, peak power output; W, watts. *Between group or pre-post statistical difference (P<0.05).
